# Complete genome sequence of the actinomycete *Actinoalloteichus hymeniacidonis* type strain HPA 177^T^ isolated from a marine sponge

**DOI:** 10.1186/s40793-016-0213-3

**Published:** 2016-12-20

**Authors:** Lena Schaffert, Andreas Albersmeier, Anika Winkler, Jörn Kalinowski, Sergey B. Zotchev, Christian Rückert

**Affiliations:** 1 Technology Platform Genomics, CeBiTec, Bielefeld University, Bielefeld, Germany; 2 Department of Pharmacognosy, University of Vienna, 1090 Vienna, Austria; 3 Sinkey Lab, Department of Biology, Massachusetts Institute of Technology, Cambridge, USA

**Keywords:** *Actinoalloteichus*, Strictly aerobic, Non-motile, Gram-positive, Non-acid-fast, Branching vegetative hyphae, Spore forming, Secondary metabolite biosynthesis gene clusters

## Abstract

*Actinoalloteichus hymeniacidonis* HPA 177^T^ is a Gram-positive, strictly aerobic, black pigment producing and spore-forming actinomycete, which forms branching vegetative hyphae and was isolated from the marine sponge *Hymeniacidon perlevis*.

Actinomycete bacteria are prolific producers of secondary metabolites, some of which have been developed into anti-microbial, anti-tumor and immunosuppressive drugs currently used in human therapy. Considering this and the growing interest in natural products as sources of new drugs, actinomycete bacteria from the hitherto poorly explored marine environments may represent promising sources for drug discovery.

As *A. hymeniacidonis*, isolated from the marine sponge, is a type strain of the recently described and rare genus *Actinoalloteichus*, knowledge of the complete genome sequence enables genome analyses to identify genetic loci for novel bioactive compounds. This project, describing the 6.31 Mbp long chromosome, with its 5346 protein-coding and 73 RNA genes, will aid the *Genomic Encyclopedia of Bacteria and Archaea* project.

## Introduction

Strain HPA 177^T^ is the type strain of the species *Actinoalloteichus hymeniacidonis*, it was isolated from the marine sponge *Hymeniacidon perlevis* at the intertidal beach of Dalian, Yellow Sea, North-China, during investigation of its actinomycete diversity [1].

Members of the diverse order *Actinomycetales* are a major source of a variety of novel bioactive and possibly pharmaceutically important compounds and drugs, such as anticancer agents [2–4], antibiotics [5, 6] and also other industrially relevant molecules and enzymes with diverse biological activities [5, 7]. Especially marine actinomycetes became a focus of research since they have evolved the greatest genomic and metabolic diversity and are auspicious sources of novel secondary metabolites and enzymes [5, 7–9].

The comparison of the complete genome sequences of members of the rare genus *Actinoalloteichus* might unravel unknown gene clusters dedicated to the biosynthesis of such molecules as bioactive secondary metabolites and enzymes. This has already been demonstrated for the genomes of strains belonging to closely related genera, such as *Kutzneria*, *Saccharomonospora*, *Crossiella* *,* *Kibdelosporangium,* and *Streptoalloteichus* [10–19].

## Organism information

### Classification and features

The genus *Actinoalloteichus* was established by Tamura et al. (2000) on the basis of morphological, physiological, chemotaxonomic and phylogenetic criteria. The genus contains Gram-positive, non-acid-fast, aerobic organisms with branching vegetative hyphae [20]. The aerial mycelium of *Actinoalloteichus* develops straight spore chains [20]. According to 16S rDNA gene sequence analysis *Actinoalloteichus* is part of the family *Pseudonocardiaceae*, suborder *Pseudonocardineae*, order *Actinomycetales*, class *Actinobacteria* [20, 21] (Table 1). It differs from other genera of its family by its morphological characteristics, fatty acid components and its non-motility [20].

**Table 1:** Classification and general features of *Actinoalloteichus hymeniacidonis* HPA 177^T^ according to the MIGS recommendations [46]

MIGS ID	Property	Term	Evidence code^a^
	Classification	Domain *Bacteria*	TAS [47]
		Phylum ‘*Actinobacteria’*	TAS [48]
		Class *Actinobacteria*	TAS [21]
		Order *Actinomycetales*	TAS [49, 50]
		Suborder *Pseudonocardianeae*	TAS [51]
		Family *Pseudonocardiaceae*	TAS [51, 52]
		Genus *Actinoalloteichus*	TAS [20]
		Species *Actinoalloteichus hymeniacidonis*	TAS [1]
		Type-strain HPA177^T^ (DSM 45092 = CGMCC 4.2500 = JCM 13436)	TAS [1]
	Gram stain	positive	TAS [1]
	Cell shape	branching hyphae	TAS [1]
	Motility	non-motile	NAS
	Sporulation	straight spores in aerial mycelia	TAS [1]
	Temperature range	mesophile (15–45 °C)	TAS [1]
	Optimum temperature	not reported	
	pH range, optimum	not reported	
	Carbon source	fructose, glucose, maltose, mannitol, mannose, xylose, rhamnose, sucrose, sorbitol, citrate	TAS [1]
MIGS-6	Habitat	Microbiological community of the intertidal marine sponge *Hymeniacidon perlevis*	TAS [1]
MIGS-6.3	Salinity	not reported	
MIGS-22	Oxygen requirement	Aerobic	TAS [1]
MIGS-15	Biotic relationship	Commensal	TAS [1]
MIGS-14	Pathogenicity	non-pathogen	NAS
MIGS-4	Geographic location	China: inter-tidal beach of Dalian, Yellow Sea	TAS [1]
MIGS-5	Sample collection time	not reported	
MIGS-4.1	Latitude	38°52′ N	TAS [1]
MIGS-4.2	Longitude	121°41′ E	TAS [1]
MIGS-4.4	Altitude	not reported	

^a^Evidence codes - *TAS* Traceable Author Statement (i.e., a direct report exists in the literature), *NAS* Non-traceable Author Statement (i.e., not directly observed for the living, isolated sample, but based on a generally accepted property for the species, or anecdotal evidence). These evidence codes are from the Gene Ontology project [53]

The genus *Actinoalloteichus* currently contains only five known species. Besides *Actinoalloteichus hymeniacidonis* HPA 177^T^ the other currently known members are the halophilic *Actinoalloteichus hoggarensis* [22], *Actinoalloteichus nanshanensis* *,* isolated from the rhizosphere of a fig tree [23], the soil bacterium *Actinoalloteichus spitiensis* [24] and *Actinoalloteichus cyanogriseus* *,* the type species of the genus isolated from a soil sample collected from the Yunnan province of China [20].

**Fig. 1:**
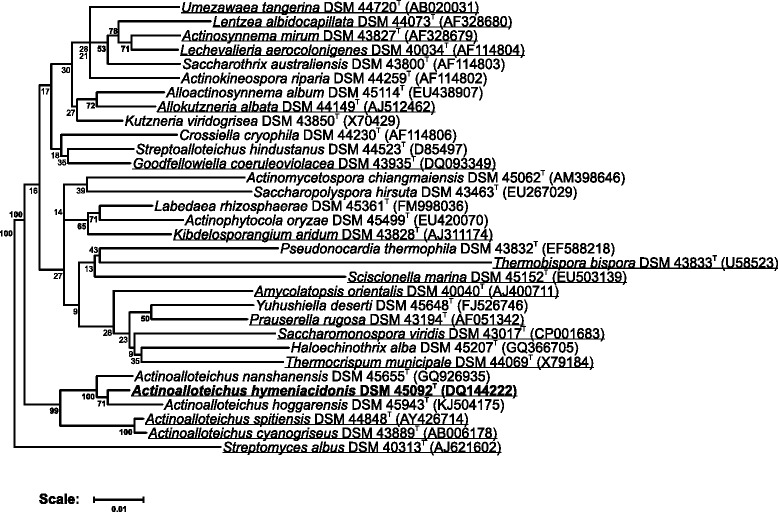
Phylogenetic tree highlighting the position of *A. hymeniacidonis* HPA 177^T^ (given in *bold*) relative to type strains of other species within the genus *Actinoalloteichus* and related genera of the family *Pseudonocardiaceae*. The tree uses sequences aligned by the RDP aligner. Using the Jukes-Cantor corrected distance model, a distance matrix is constructed based on alignment model positions without the use of alignment inserts, using a minimum comparable position of 200. The tree is built with RDP Tree Builder, which utilizes Weighbor [54] with an alphabet size of 4 and length size of 1000. The building of the tree also involves a bootstrapping process repeated 100 times to generate a majority consensus tree [55]. *Streptomyces albus* DSM 40313^T^ was used as the root organism. Species for which a complete or draft genome sequence is available are *underlined*

A representative 16S rRNA sequence of *A. hymeniacidonis* HPA 177^T^ was compared to the Ribosomal Database Project database [25] confirming the initial taxonomic classification. On the basis of the 16S rDNA, *A. hymeniacidonis* shows highest similarity to *A. hoggarensis* AH97^T^ (99.2%) and *A. nanshanensis* NEAU119^T^ (98.3%). Together with *A. spitiensis* DSM 44848 ^T^ (96.8%) and *A. cyanogriseus* IFO 14455^T^ (96.4%), they form a distinct clade within the family *Pseudonocardiaceae*. Figure 1 shows the phylogenetic neighborhood of *A. hymeniacidonis* in a 16S rRNA gene based tree.

**Fig. 2:**
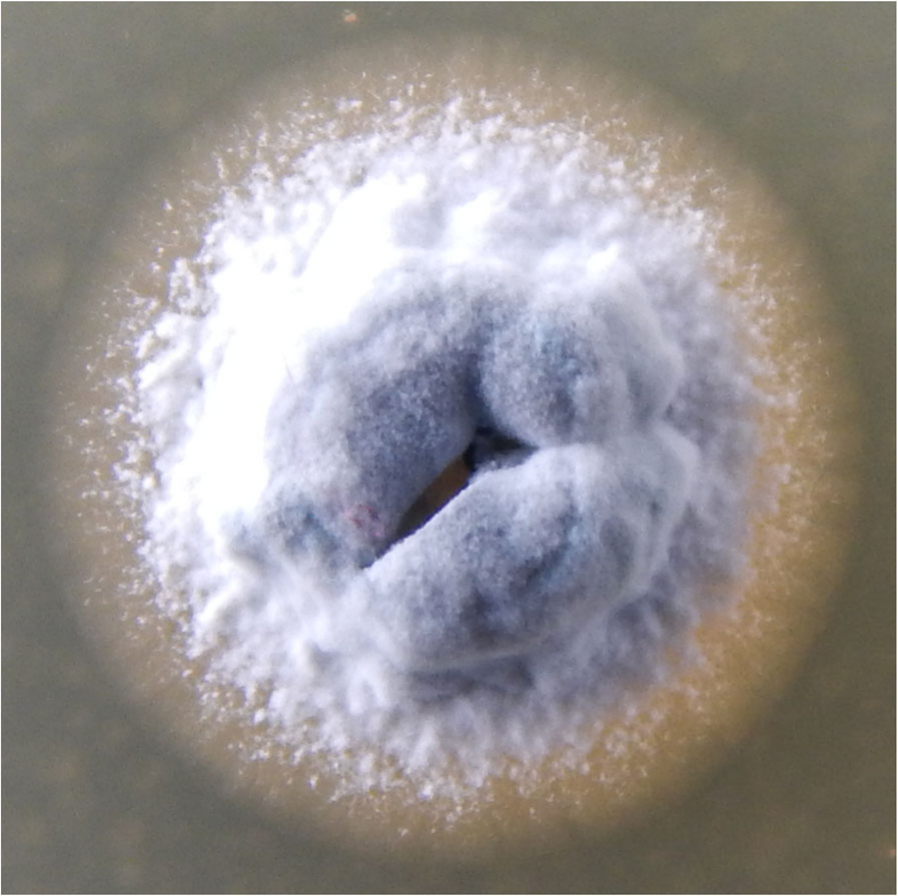
Colony of *A. hymeniacidonis* HPA 177^T^ grown at 28 °C for 8 days on ISP2 agar medium prepared with artificial sea water

*A. hymeniacidonis* HPA 177^T^ forms branching vegetative hyphae (Fig. 2), which are grey to black in color and tend to fragment after 3 weeks of cultivation (1). The aerial hyphae develop spores of a dimension of 0.6 × 0.8 μm [1]. HPA 177^T^ is strictly aerobic and non-motile [1]. Growth of *A. hymeniacidonis* was shown at temperatures between 15 and 45 °C (optimal growth between 20 and 37 °C) [1]. HPA 177^T^ can utilize fructose, glucose, maltose, mannitol, mannose, xylose, rhamnose, sucrose, sorbitol, sodium citrate, casein, or starch as carbon sources, but not arabinose, inositol, and raffinose [1] (Table 1). It grows well on yeast extract/malt extract agar or oatmeal agar and produces a black soluble pigment when growing on yeast extract/malt extract agar as well as on peptone/yeast extract/iron agar [1]. It has been shown that the strain grows faster on ISP2 agar media prepared with 50% of artificial sea water, which, considering the source of isolation, probably reflects an adaptation to the marine environment. Urea is not decomposed by *A. hymeniacidonis*, and this strain shows neither hydrolysis of aesculin or hippurate, nor utilization of calcium malate, sodium oxalate, or sodium succinate nor reduction of nitrate [1].

#### Chemotaxonomic data

The cell wall of *A. hymeniacidonis* contains diaminopimelic acids (A_2_pm) [1]. The major menaquinone is MK-9(H_4_) (64%), followed by MK-9(H_6_) (23%) and MK-9(H_8_) (12%).

The phospholipids were shown to be mainly composed of phosphatidylethanolamine, phosphatidylglycerol, phosphatidylinositol, phosphatidylinositol mannoside as well as of some other glucosamine containing phospholipids of unknown structure as diagnostic polar lipids [1]. *A. hymeniacidonis* does not contain mycolic acids [1].

The cellular fatty acids are mainly composed of anteiso pentadecanoic acid (C_15:0_ anteiso) (20%), cis-8-heptadecenoic acid (C_17:1_ *ω8c*) (19%), isopalmitic acid (C_16:0_ iso) (16%), heptadecanoic acid (C_17:0_) (11%) and other fatty acids occurring in lower amounts [1]. Galactose, glucose, mannose, and ribose are whole cell sugars of HPA 177^T^ [1].

## Genome sequencing information

### Genome project history

Due to the increasing interest in exploiting new and rare actinomycetes as new sources of novel secondary metabolites [5], *Actinoalloteichus hymeniacidonis* HPA 177^T^, a member of the rare genus *Actinoalloteichus* [20], was selected for sequencing. While not being part of the GEBA project [26], sequencing of the type strain will aid the GEBA effort. The genome project is deposited in the Genomes OnLine Database [27] and the complete genome sequence is deposited in GenBank. A summary of the project information is shown in Table 2.

**Table 2:** Genome sequencing project information

MIGS ID	Property	Term
MIGS-31	Finishing quality	Finished
MIGS-28	Libraries used	Nextera DNA Sample Prep Kit, Nextera Mate Pair Sample Prep Kit
MIGS-29	Sequencing platforms	Illumina MiSeq
MIGS-31.2	Fold coverage	159.00×
MIGS-30	Assemblers	Newbler version 2.8
MIGS-32	Gene calling method	GeneMark, Glimmer
	Locus Tag	TL08
	GenBank ID	CP014859
	GenBank Date of Release	September 28, 2016
	GOLD ID	Gp01114707
	NCBI project ID	PRJNA273752
MIGS-13	Source material identifier	DSM 45092
	Project relevance	Industrial, GEBA

### Growth conditions and DNA isolation

*A. hymeniacidonis* HPA 177^T^ was grown aerobically in 50 ml 3% TSB medium (Oxoid, UK) in 250 mL baffled flasks at 28 °C, 250 rpm. Genomic DNA was isolated using Wizard Genomic DNA Purification Kit (Promega, USA) from ~2 g of mycelium (wet weight) using the manufacturer’s protocol with the following modification. The clarified lysate prior to precipitation of DNA with isopropanol was extracted once with ½ volume of a 1:1 mixture of phenol/chloroform (pH 8.0).

### Genome sequencing and assembly

Two libraries were prepared: a WGS library using the Illumina-Compatible Nextera DNA Sample Prep Kit (Epicentre, WI, U.S.A.) and a 6 k MatePair library using the Nextera Mate Pair Sample Preparation Kit, both according to the manufacturer’s protocol. Both libraries were sequenced in a 2× 250 bp paired read run on the MiSeq platform, yielding 4,594,541 total reads, providing 159.00× coverage of the genome. Reads were assembled using the Newbler assembler v2.8 (Roche). The initial Newbler assembly consisted of 31 contigs in five scaffolds, with a total of 50 contigs larger than 100 bp. Analysis of the five scaffolds revealed three to make up the chromosome and the remaining two containing the three copies of the RRN operon.

The Phred/Phrap/Consed software package [28–31] was used for sequence assembly and quality assessment in the subsequent finishing process, gaps between contigs were closed by manual editing in Consed (for repetitive elements).

### Genome annotation

Gene prediction and primary annotation were done using the IMG ER pipeline [32]. Additionally, genes were identified using GeneMark [33], GLIMMER [34], and Prodigal [35]. For annotation, BLAST searches against the NCBI Protein Clusters Database [36] were performed and the annotation was enriched by searches against the Conserved Domain Database [37] and subsequent assignment of coding sequences to COGs. Non-coding genes and miscellaneous features were predicted using tRNAscan-SE [38], Infernal [39], RNAMMer [40], Rfam [41], TMHMM [42], and SignalP [43].

## Genome properties

**Fig. 3:**
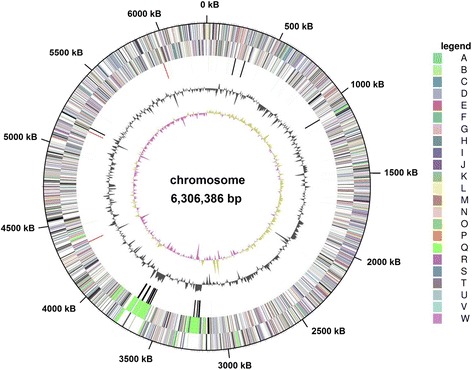
Graphical map of the chromosome of *A. hymeniacidonis* HPA 177^T^. From the outside to the center: Genes on forward strand (colored by COG categories), genes on reverse strand (colored by COG categories), RNA genes (tRNAs *green*, rRNAs *red*, other RNAs *black*), G+C content, G+C skew

The genome includes one circular chromosome of 6,306,386 bp (68.08% G+C content) (Fig. 3). Among a total of 5425 predicted genes, 5346 are protein coding genes. 4068 (74.90%) of the protein coding genes were assigned a putative function, the remaining were annotated as hypothetical proteins. The properties and the statistics of the genome are summarized in Tables 3 and 4, and the circular plot is shown in Fig. 3.

**Table 3:** Genome Statistics

Attribute	Value	% of total^a^
Genome size (bp)	6,306,386	100.00
DNA coding (bp)	5,516,402	87.47
DNA G+C (bp)	4,293,157	68.08
DNA scaffolds	1	100.00
Total genes	5425	100.00
Protein-coding genes	5346	98.54
RNA genes	73	1.34
Pseudo genes	6	0.11
Genes with internal clusters	753	13.86
Genes with function prediction	4068	74.90
Genes assigned to COGs	3329	61.30
Genes with Pfam domains	4327	79.67
Genes with signal peptides	381	7.02
Genes with transmembrane helices	1271	23.40
CRISPR repeats	15	

^a^The total is based on either the size of the genome in base pairs or the total number of total genes in the annotated genome

**Table 4:** Number of genes associated with the general COG functional categories

Code	value	% age	Description
J	206	5.33	Translation, ribosomal structure and biogenesis
A	1	0.03	RNA processing and modification
K	439	11.36	Transcription
L	109	2.82	Replication, recombination and repair
B	1	0.03	Chromatin structure and dynamics
D	33	0.85	Cell cycle control, cell division, chromosome partitioning
V	150	3.88	Defense mechanisms
T	184	4.76	Signal transduction mechanisms
M	159	4.11	Cell wall/membrane biogenesis
N	7	0.18	Cell motility
U	29	0.75	Intracellular trafficking and secretion, and vesicular transport
O	136	3.52	Posttranslational modification, protein turnover, chaperones
Z			Cytoskeleton
W	4	0.1	Extracellular structures
C	213	5.51	Energy production and conversion
G	348	9	Carbohydrate transport and metabolism
E	334	8.64	Amino acid transport and metabolism
F	94	2.43	Nucleotide transport and metabolism
H	255	6.6	Coenzyme transport and metabolism
I	181	4.68	Lipid transport and metabolism
P	204	5.28	Inorganic ion transport and metabolism
Q	190	4.91	Secondary metabolites biosynthesis, transport and catabolism
R	450	11.64	General function prediction only
S	135	3.49	Function unknown
X	4	0.1	Mobilome: prophages, transposons
-	2102	38.7	Not in COGs

## Insights from the genome sequence

### Gene clusters for biosynthesis of secondary metabolites

So far, there have been no reports on isolation of secondary metabolites from *A. hymeniacidonis* HPA 177^T^. However, keeping in mind that all actinomycete genomes sequenced so far contain SMBGCs, the genome of strain HPA 177^T^ was analyzed for their presence using the online version of software antiSMASH 3.0.4 [44]. The results of the analysis were manually curated to confirm or edit borders of the clusters, identify closest homologues in the databases based on BLAST search (Table 5), and to gain a more detailed insight into the biosynthesis of the corresponding compound. In total, 25 SMBGCs were identified, 11 of which appeared to be unique at the time of analysis and based on the public database searches. This conclusion was based on the unique composition of the core genes in the clusters encoding scaffold-building enzymes, and in some cases, such as stand-alone terpene cyclase or type III polyketide synthase genes, on low (below 60%) identity of their products to proteins in the NCBI database. Based on this analysis, it seems possible that *A. hymeniacidonis* HPA 177^T^ has the genetic capacity to produce novel compounds some of which, e.g. peptide-polyketide hybrids, terpenoids, and unique lassopeptides, may represent bioactive metabolites suitable for drug development. Given its habitat, *A. hymeniacidonis* might be the real source of secondary metabolites that are thought to originate from its host sponge, comparable to. e.g. *Theonella* *swinhoi* and *Entotheonella* sp. [45]. The knowledge on the SMBGCs and their putative products will assist in identification of the corresponding compounds, and may pave the way to biosynthetic engineering toward generation of new analogues.

**Table 5:** Secondary metabolite biosynthesis gene clusters identified in the genome of *Actinoalloteichus hymeniacidonis* DSM 45092 using antiSMASH 3.0.4 software followed by manual curation

No	Cluster type	Presence in another bacterium^#^	Putative product
1	Ectoine	*Saccharopolyspora rectivirgula* DSM 43113	Ectoine
2	NRPS-PKSI	*Nonomuraea candida* DSM 45086	NRS peptide-polyketide hybrid
3	Ladderane	*Saccharomonospora viridis* DSM 43017	Ladderane
4	NRPS-PKSI	-	NRS peptide-polyketide hybrid
5	Ectoine	multiple *Actinoalloteichus* spp*.*	Ectoine
6	Lassopeptide	-	Lassopeptide
7	Terpene	*Kribbella flavida* DSM 17836	Terpenoid
8	PKSII	-	Aromatic polyketide
9	Terpene	-	Terpenoid
10	Siderophore	*Saccharomonospora paurometabolica* YIM 90007	Siderophore
11	Terpene	*Actinosynnema mirum* DSM 43827	Carotenoid
12	PKSIII	-	Stilbene-like polyketide
13	NRPS-PKSI	*Streptomyces* sp. NTK 937	Polycyclic tetramate macrolactam
14	NRPS	*Streptomyces* sp. SirexAA-E	Coelibactin
15	PKSI	-	34-membered macrocyclic lactone
16	NRPS-PKSI	*Streptomyces bingchenggensis* BCW-1	NRS peptide-polyketide hybrid
17	Terpene	-	Terpenoid
18	NRPS	-	NRS peptide
19	PKSI	*Saccharomonospora xinjiangensis* XJ-54	Glycosylated polyene macrolide
20	NRPS	-	Mannopeptimycin-like NRS peptide
21	PKSI	*Amycolatopsis nigrescens* CSC17Ta-90	Hygrocin-like polyketide
22	Oligosaccharide	*Nocardiopsis kunsanensis* DSM 44524	Oligosaccharide
23	Butyrolactone	-	Butyrolactone
24	Siderophore	-	Siderophore
25	PKSII	*Microbispora* sp. ATCC PTA-5024	Aromatic polyketide

Notes: *NRS* non-ribosomally synthesized. Shaded cells show potentially unique gene clusters. ^#^Presence in other bacteria based on the publically available data as of January 27, 2016

## Conclusion

The genome sequence of *A. hymeniacidonis* HPA 177^T^ represents the first genome of the *A. hoggarensis*/*A. hymeniacidonis*/*A. nanshanensis* subgroup, the first complete genome of this genus as well as the first of a marine species of this genus. As such, it will be a useful basis for future genome comparisons. The presence of 25 SMBGCs indicates a great potential for secondary metabolite production, either by heterologous expression in suitable hosts or by activating the clusters by genetic engineering.

## Abbreviations

CeBiTec: Center for Biotechnology
GEBA: *Genomic Encyclopedia of Bacteria and Archaea*
SMBGC: Secondary metabolite biosynthesis gene cluster
